# Structuring and enriching the rearing environment in conventional broiler chicken production: effects on behavioral indicators, emotional states, and cecal microbiota composition^☆^

**DOI:** 10.1016/j.psj.2025.105663

**Published:** 2025-08-06

**Authors:** M. Guinebretière, M. Guyard-Nicodème, F. Mocz, L. Calandreau, A. Scheubel, J.P. Moysan, M. Chemaly, A. Keita, L. Warin

**Affiliations:** a Epidemiology, Health and Welfare Unit, Ploufragan-Plouzané-Niort Laboratory, French Agency for Food, Environmental and Occupational Health & Safety (ANSES), Ploufragan, France; b Unit of Hygiene and Quality of Poultry and Pork Products, Ploufragan-Plouzané-Niort Laboratory, French Agency for Food, Environmental and Occupational Health & Safety (ANSES), Ploufragan, France; c INRAE, CNRS, IFCE, Université de Tours, Centre Val-de-Loire UMR Physiologie de la Reproduction et des Comportements, 37380 Nouzilly, France; d Avian Experimental Unit, Ploufragan-Plouzané-Niort Laboratory, French Agency for Food, Environmental and Occupational Health & Safety (ANSES), Ploufragan, France; e Technical Institute for Poultry (ITAVI), 37380 Nouzilly, France

**Keywords:** Welfare, Emotional reactivity, Behavior, Positive welfare, Environmental enrichment

## Abstract

Providing a variety of elements in the rearing environment may offer a promising way to transition conventional broiler production towards more animal-friendly production systems. This study aimed to investigate the effect of a complex and structured rearing environment on broiler welfare by comparing three complex enriched rooms (**E**) with visual cues and multiple elements for resting or exploration, to three control rooms (**C**) with minimal enrichments. Each room housed 2450 Redbro chickens and received natural light. We evaluated how the rearing environment influenced chicken behavior, emotional state, and the cecal microbiota composition at the end of the rearing period. E chickens stood, foraged and moved more often than C chickens (e.g. 38% vs. 19% walking; 4% vs. 1% foraging, *P* < 0.001), while dustbathing frequency did not differ. In group-based tests, E chickens were more likely to approach and peck at a novel object or human (*P* < 0.05), suggesting reduced fearfulness and increased curiosity. In the detour test, E chickens exited the U-shaped area more frequently (*P* < 0.001) and vocalized less (*P* = 0.004), indicating greater exploratory motivation and possibly better spatial cognition. Microbiota analysis revealed no differences in alpha diversity, but beta diversity differed significantly between treatments (*P* < 0.001). E chickens had higher relative abundances of *Bacteroidota*, while C chickens had more *Bacillota*. Overall, the complex environment promoted natural behaviors, reduced emotional reactivity, and altered gut microbiota composition, supporting its potential to improve broiler welfare under semi-commercial conditions.

## Introduction

For over five decades, the conventional broiler industry has focused on optimizing growth performance, feed efficiency, and breast muscle size to meet the rising global demand for poultry meat (Tallentire et al., 2016). This progress has been largely driven by genetic selection, along with improvements in resources and management practices. However, these advances in production also resulted with unintended consequences. Genetic selection has led to morphological, physiological, and behavioral changes in chickens that have often resulted in a deterioration of key welfare indicators, such as physical activity levels (Hartcher and Lum, 2020; Riber and Wurtz, 2024). In addition, standardized rearing environments, designed to maximize genetic potential and control health risks, restrict the expression of certain behavioral needs such as exploratory behavior, perching or dustbathing (Wallenbeck et al., 2016; Dixon, 2020; Dawson et al., 2021), thereby compromising overall animal welfare. While progress has been made in improving health and optimizing housing conditions, addressing welfare issues has emerged as a critical challenge for future development.

The conditions in which animals are kept significantly influence their welfare in multiple ways. Animal welfare encompasses both mental and physical well-being, shaped by the fulfillment of physiological and behavioral needs. Therefore, welfare is not solely defined by physical health, but also includes an animal’s capacity to perceive, interpret, and respond to its environment, an aspect closely linked to its emotional state (ANSES, 2018). Environmental enrichment has been shown to enhance broiler welfare by promoting the expression of such as foraging and perching (Riber et al., 2018; Vasdal et al., 2019). In addition, recent research has shown that broilers raised in enriched environments are more active and exhibit reduced startle reactivity (Ross et al., 2020), which can enhance both their physical and emotional health. Other studies emphasize the role of environmental enrichment in supporting cognitive and emotional development (Tahamtani et al., 2018), thereby improving the animals' ability to cope with stress and experience positive emotional states. For instance, one study found that a greater proportion of chicks reared in enriched conditions maintained an optimistic bias (i.e. interpreted ambiguous situations more positively) even after exposure to stressors, compared to those raised in barren environments (Zidar et al., 2018). This may enhance their adaptability to varying rearing conditions and reduce susceptibility to negative affective states (Campbell and Lee, 2021). In contrast, barren environments, particularly during early life stages, may have long-lasting adverse effects on behavior, adaptability, and resilience to stress, potentially leading to issues such as feather pecking and increased fearfulness (Rodenburg and de Haas, 2016).

Furthermore, several studies have demonstrated that enriched environments contribute to improved gut microbiota, which is closely linked to overall health and stress resilience (Apajalahti, 2005; Oakley and Kogut, 2016; Sugiharto, 2016). Evidence suggests that enrichments such as the provision of perches, litter, and a complex rearing environment can promote healthier microbiota (characterized by increased gut microbial alpha diversity, improved gut microbial colonization and development of gut flora). This, in turn, supports optimal growth (Torok et al., 2009) and a more robust immune response (Yan et al., 2020; de Jong et al., 2022). Consequently, animal welfare standards related to housing, health, and nutrition may be closely associated to the composition of the cecal microbiota (Di Marcantonio et al., 2022).

Our research aims to generate valuable insights for transitioning conventional broiler production systems toward models that enhance animal welfare without incurring substantial economic costs. Environmental enrichment appears to be a promising strategy, as it enables broilers to express their behavioral needs. Moreover, implementing enrichment measures is often less costly than other interventions, such as reducing stocking density or slowing growth rate (Méda et al., 2024). To this end, we evaluated the effects of a complex and enriched indoor rearing environment on broiler welfare under semi-commercial conditions. Unlike previous studies that often introduced isolated enrichment items into otherwise barren environments, our approach involved structuring the entire space to promote both physical and mental stimulation. Complex and structured environments may enhance spatial cognitive abilities, as demonstrated in laying hens by Gunnarsson et al. (2000). Our intention was to test this approach under conditions that are transferable to commercial farming, i.e., using large groups of animals in semi-commercial settings rather than under highly experimental conditions with small samples sizes. The enriched environment included a variety of elements designed to structure the space and provide opportunities for physical activity, exploration, and cognitive engagement. This fostered the agency of birds (i.e., their capacity to act intentionally upon themselves, others, and their environment) by offering them meaningful choices between areas for rest and exploration. It was compared to a control environment with minimal resources.

We hypothesized that such an environment would promote positive experiences, referred to as positive animal welfare (Rault et al., 2025), including increased expression of natural behaviors, improved emotional states, and enhanced cognitive abilities.

To assess this, we conducted a multidisciplinary evaluation focusing on three main dimensions of welfare:

- Behavioral expression, through observations of locomotion and foraging.
- Emotional state and spatial cognition, assessed via standardized behavioral tests (novel object, human approach, novel environment, and detour test), which require no prior training and minimize the influence of physical ability (Norman et al., 2019).
- Health-related welfare, by analyzing cecal microbiota composition as a potential physiological indicator of stress resilience and gut health.

Additionally, we included growth performance indicators to evaluate the feasibility of implementing such enriched environments in commercial production systems.

## Materials and methods

### Ethics statement

The housing, management, and experimental procedures complied with European legislation on the protection of animals used for scientific purposes (EU Directive 2010/63/EU) and were approved by the laboratory’s Animal Welfare Committee (No. d-22-745-1, Opinion no. 2022-09-13-02).

### Housing and experimental scheme

A total of 14,700 one-day-old Redbro broilers were delivered to the ANSES Avian Experimental Unit in Ploufragan, France. The chicks (sexes not distinguished) were distributed across six rooms, each measuring 162 m², within the same experimental broiler facility (2,450 chicks per room, corresponding to a stocking density of 15 chicks/m²). Birds were reared until 43 days of age, reaching an average body weight of approximately 2.2 kg, which corresponded to a final stocking density of around 33 kg/m² (accounting for a 3.5 % mortality rate).

**Fig. 1:**
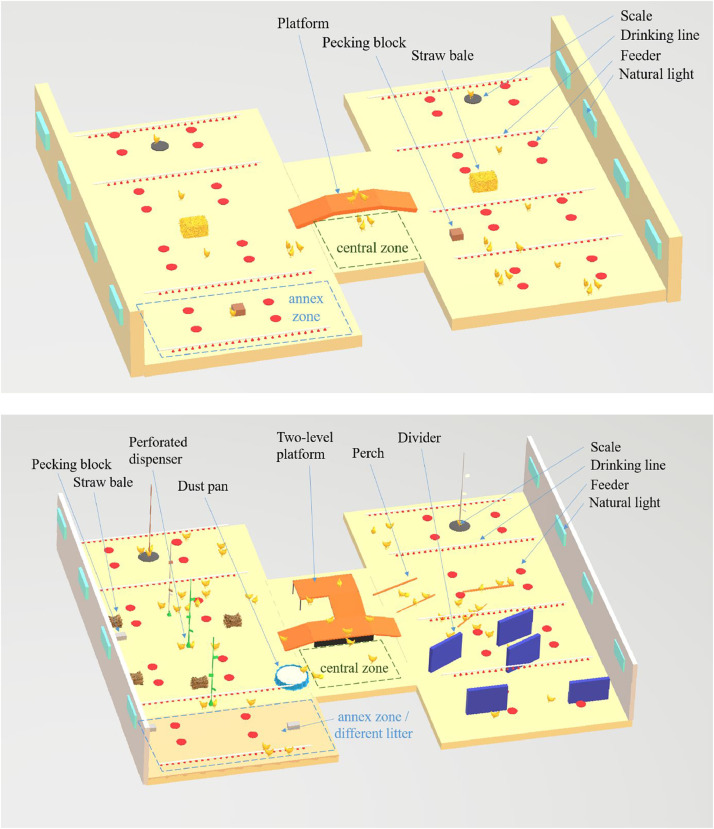
Control environment (C) room (above) and enriched environment (E) room (below) showing the position of the window, drinking line, feeders, automatic scales, enrichments and observation zones (central and annex). In E room, right-hand side was more intended for resting behaviors, while left-hand side was more intended for active behaviors.

Three control rooms (**C**) had a few enrichments (Fig. 1), corresponding to an improved standard rearing system relative to the regulatory minimum (Directive 2007/43/EC):

- one platform (30 cm high with access ramps on either side, totaling a surface area of 4 m², Josse, Montauban-De-Bretagne, France);
- two pecking blocks composed of minerals (oyster shells) and organic inclusions (extruded linseed, brewer's yeast, extruded wheat, and a mixture of aromatic plants) weighing 8 kg and 12 cm high × 19 cm long × 19 cm wide (Pikee Bloc, Wisium, Saint-Nolff, France);
- and two firmly pressed straw bales (15 kg, 30 cm high × 50 cm long × 30 cm wide (Desialys, Paris, France).

The three complex enriched rooms (**E**) had visual markers to help animals locate (colored markings on the walls, on the weighing chains, and feed hopper chains) and various enrichments divided into two main areas (Fig. 1). The first area was dedicated to resting with

- a two-level platform (30 and 50 cm high with access ramps on either side and between the two levels, totaling a surface area of 7 m², Josse, Montauban-De-Bretagne, France), which helped to structure the space and meet the need for high perches, and resting;

- fringes were added along the first level of the platform to create a dark space below, providing a sheltered area;

- four linear perches (10 cm high × 150 cm long × 10 cm wide, Lubing, Sailly-Sur-La-Lys, France), with a mushroom-shaped section appropriate for chicken feet, offering stability and grip when perching;

- five vertical dividers (50 cm high × 100 cm long × 5 cm wide, Josse, Montauban-De-Bretagne, France) to help structure the space and meet the need for gregarious resting and shelter.

The other area was dedicated to exploration, with a focus on scratching, pecking, dustbathing, and offering:

- perforated dispensers for pecking, filled successively with oats, corn, wheat, or grit (three suspended dispensers, 15 cm high x 8.5 cm wide, Omlet, Banbury, UK and three foraging balls placed on the floor, 7 cm in diameter, Chicken Fun, Savic, Ferme de Beaumont, Eu, France),
- one dust pan filled with wood dust (21 cm high × 77 cm long × 87 cm wide),
- three pecking blocks (similar to the ones in C rooms)
- four straw bales (similar to the ones in C rooms),
- one 18 m² area with wood shavings (called the "**annex zone**").

In these E rooms, staff knocked on the door before entering, to provide an audible signal before entry.

All the rooms were illuminated by natural daylight from 08:00 to 19:00 through eight windows (90 cm × 30 cm), supplemented as needed by artificial lighting to ensure a minimum of 30 lux from 06:00 to 23:59 starting from day 6. Sawdust litter was provided in accordance with standard management practices (1.3 kg/m² on a concrete floor). Rearing parameters (feeding, lighting program, temperature, ventilation) and vaccination followed conventional commercial specifications. All birds were fed *ad libitum* with diets formulated to meet the nutritional requirements of the strain.

### Data collection

Use of Enrichments. To evaluate the use of the different enrichments items in the E and C rooms, photographs of the enrichments (platforms, perches, straw bales, dividers, dust pan) were taken daily at the same time (7 am), before the animal handler entered the rooms for routine observations. The number of chickens using the enrichments (either perched on or positioned alongside them) was counted retrospectively by a single observer. Pecking blocks were individually weighed on D42. Use was estimated by calculating the difference between the initial and final weights, which reflects both actual ingestion, as well as the dispersion of particles into the litter due to pecking, scratching and perching, without the possibility of distinguishing between them. Each morning, the remaining quantity of pecking material in the perforated dispensers (suspended and on the floor) was visually assessed.

Comparisons between the C and E treatments were based on several criteria: locomotion, foraging, and dustbathing behaviors, emotional state; spatial cognitive abilities; and cecal microbiota composition. In addition, selected health and growth performance indicators were also compared between the two treatments. They criteria are detailed in the sections below.

Locomotion, Foraging and Dustbathing. Birds behaviors were observed in each room, within a designated “central zone” (a 6 m² area free of enrichments, drinkers and feeders, see Fig. 1). Observations were conducted during 3-minute periods, repeated three times a day at four different ages, resulting in a total of 12 observation sessions per room. As it was not feasible to monitor all behaviors simultaneously, locomotor behaviors were observed in the morning by two persons, while dustbathing and foraging behaviors were recorded in the afternoons by a single observer. These time slots were selected based on prior observations indicating that they best correspond to the natural expression of these behaviors in laying hens (Guinebretière et al., 2014, 2015).

- Locomotor behavior was assessed at approximately 8 am, 10.30 am and 12 noon, on D15, D20, D27, and D37. Observations were conducted by two observers positioned outside the pen, focusing on a predefined central area. After a 4-minute habituation period, scan sampling was used to record the number of chickens present in the area and, among them, the number of chickens classified as “Standing”, both before and after a 3-minutes observation period. During the 3-minutes interval, one observer recorded all the “Running” events, while the second observer simultaneously counted all the “Walking” and “Crossing the area” events. A detailed description of the recorded behaviors is provided in Table 1.

**Table 1:** Ethogram used for the assessment of behavior (inspired from Bergmann et al. (2017)).

Behavior event	Operational definition
Standing	Chicken stands upright on both legs
Running	Chicken moves more than 3 steps at a fast pace, and may flap its wings
Walking	Chicken moves more than 3 steps at a slow pace
Crossing the area	Chicken enters and leaves the observed area while running
Foraging	Chicken stands in an upright position with both feet on the ground, uses both feet alternatively to scratch at the ground, and/or lowers its head from time to time to peck at or move litter material
Dustbathing	Chicken pecks and scratches at the litter material, then crouches down in the substrate, and follows an organized sequence of behavior patterns (crouching, stirring the litter and shaking)
- Dustbathing and foraging behaviors were observed at approximately 2 pm, 3:30 pm and 6 pm, on the following days (D16, D21, D28, and D38). A single observer, positioned outside the pen, conducted observations in the central zone following a 4-minute habituation period. Using scan sampling before and after a 3-minute observation period, the number of chickens present in the area was recorded. During the 3-minute interval, all the “Dustbathing” and “Foraging” events were counted (see Table 1). In addition to the central zone, the same observer used the same protocol to record the number of chickens present, and the number of “Dustbathing” and “Foraging” events in the entire Annex zone in the E rooms (18 m²), as well as in the corresponding area in the C rooms (see Fig. 1), during the same afternoons sessions.

For “Running”, “Walking”, “Crossing the area”, “Dustbathing” and “Foraging”, all behavioral events were recorded regardless of which individual performed them. If a bird interrupted a behavior for more than 5 seconds, a new occurrence was counted. As a result, multiple behavioral events could be attributed to a single bird within the 3-minute observation period, either for the same behavior or for different behaviors.

Emotional State: Novel Environment, Reactivity and Detour Tests. A set of validated tests (novel environment (NE) and reactivity tests) (Forkman et al., 2007) was conducted, along with a detour test, to assess the birds’ cognitive abilities and various dimensions of emotional reactivity (Wichman et al., 2007). The timing of the tests was carefully planned to balance two constraints: allowing sufficient exposure to environmental conditions to influence behavior, while avoiding the reduced mobility associated with rapid growth. Logistical constraints also prevented all tests from being conducted within the same time window. The detour and NE tests were each performed once, while the reactivity tests were conducted twice. Reactivity tests were carried out at the group level, using the room as the experimental unit, which limited replication to three per treatment. Repeating these tests over time helped ensure the consistency of the results. In contrast, the detour and NE tests were performed on randomly selected samples of birds from each room (60 birds per treatment), providing sufficient statistical power for individual-level analysis.

Novel Environment Test. From D23 to D24, between 2 pm and 6 pm, 60 birds per treatment group (20 per room) underwent a NE test (de Jong et al., 2003). The test was conducted outside the rearing pens but within the rearing room to maintain a consistent ambient conditions. Each bird was placed in a corner of a square arena (100 cm high x 100 cm long x 100 cm wide) with a litter-free floor divided into four equal zones. The objective of the test was to assess general fearfulness as well as the effect of social isolation and dependence (Forkman et al., 2007). The following variables were recorded: latency to first step, latency to first zone change, number of zone changes, and number of vocalizations. Each test lasted 3 minutes. To minimize disturbance, the observer remained behind a wall with a viewing hole at eye level throughout the test.

Reactivity Tests. At D29 and D36, between 9 am and 11 am, each room was tested by a single observer for reactivity to a novel object (NO test) following the Welfare Quality® (2009). The observer gently placed a novel object (a colored can) in the center of the room. Every 30 seconds over a 5-minute period, the number of chickens approaching the object (within a 30 cm radius) and the number pecking at it were recorded. The same procedure was applied in all six rooms. Similarly, a human reactivity test (H test) was conducted at D31 and D38 using the same protocol. In this case, the novel stimulus was an unfamiliar human standing in the center of the room, and the approach zone was extended to a 1-meter radius. These NO and H tests were designed to assess different aspects of the birds' reactivity, specifically, their responses to environmental stimuli and to humans.

Detour Test. At D21–D22, between 2 pm and 6 pm, 60 birds per treatment group (20 per room) underwent a detour test (Wichman et al., 2009), conducted on one side of the rearing pen to maintain identical floor and ambient conditions. Each bird was placed in a test arena (200 cm long x 100 cm wide x 60 cm high) behind a U-shaped mesh fence. From this position, the bird could see its conspecifics in the rearing pen through the mesh but could only reach them by circumventing the fence. The objective of the test was to assess the chicken’s spatial problem-solving ability, specifically, its capacity to exit the detour area and rejoin its conspecifics. The following variables were recorded: success in exiting the U-shaped area, success in reaching their congeners, the corresponding latencies, and the number of vocalizations. The test lasted a maximum of 5 minutes or ended earlier if the chicken successfully reached its conspecifics. Early success in this test may indicate enhanced spatial cognitive abilities (Norman et al., 2019). To minimize disturbance, the observer remained behind a wall with a viewing hole at eye level throughout the test.

### Health and growth performances

Microbiota analysis. On D41, 92 male broiler chickens (46 from a single E room and 46 from a single C room) were randomly selected and euthanized (intravenous injection with Dolethal ND, Vetoquinol, Lure, France, 150 mg/kg of active ingredient: sodium pentobarbital) for cecal sampling. DNA from cecal contents was extracted using the NucleoMag Tissue Kit (Macherey-Nagel, Hoerdt, France) as described in Gloanec et al. (2022). The V3-V4 region of the 16S rRNA gene was amplified (forward primer: 5′TCGTCGGCAGCGTCAGATGTGTATAAGAGACAGCCTACGGGNGGCWGCAG3’; reverse primer: 5′GTCTCGTGGGCTCGGAGATGTGTATAAGAGACAGGACTACHVGGGTATCTAATCC-3′). Amplicons were sequenced as described in Gloanec et al. (2022) using Illumina MiSeq platform generating 2 × 300 bp paired-end reads. A total of 89 samples were successfully sequenced; three samples (one from the C group and two from the E group) were removed due to the low number of generated reads (< 6000 reads). Sequence processing was performed using FROGS (Version 4.1.0) (Escudié et al., 2018). Briefly, paired-end reads were merged using VSEARCH. Sequences were filtered by excluding those with lengths <380 and >500 nucleotides, those with ambiguous bases and those without a primer sequence at both 3′ and 5′ ends (no mismatch allowed). SWARM was used to cluster amplicon sequence variants (ASVs). VSEARCH was used to remove chimeras. ASVs were excluded when representing less than 0.005 % of the total number of sequences (Bokulich et al., 2013) and if an ASV present in one group was present in at least half of samples making up this group. Contaminating sequences were excluded using the phiX databank. Taxonomy was assigned using REFSeq database (/NCBIdb_bacteria_16S_v1.20230726).

*Body Weight, Leg Health, Breast Cleanliness and Mortality.* On D35, 150 birds per treatment group (50 birds per room, randomly selected with balanced sex rations) were assessed for body weight, pododermatitis, hock burns, gait, and breast cleanliness. Each criterion was scored on a scale from 0 to 2, with lowest scores indicating the better physical condition (see Table 2).

**Table 2:** Scoring of footpad dermatitis, hock burns, gait score and breast cleanliness (Guinebretière et al., 2024).

	Score 0	Score 1	Score 2
**Pododermatitis**	None or slight, very superficial lesions, slight discoloration on a limited area, slight hyperkeratosis or scarred skin	Moderate and severe discoloration, superficial lesions, darkened papillae	Severe dermatitis ulcers or scabs of significant size, signs of bleeding or severely swollen foot pads
**Hock burns**			
**Gait**	Normal gait, agile, with or without imbalance	Chicken walks more than 1.50 m but has difficulty in walking; limps	Chicken walks less than 1.50 m, and/or is severely lame, preventing movement
**Breast cleanliness**	Clean — no stains on the feathers — nothing stuck to the skin or feathers	A few spots on the feathers, not covering the whole breast, no litter or stuck-on droppings	Most feathers are stained, and there may be dirty litter or stuck-on droppings

For the pododermatitis, hock burn, and cleanliness scoring, each bird was gently picked up and its legs and breast were examined by a trained observer, who recorded the condition of the worst-affected leg and the cleanliness of the breast. The bird was then weighed on a scale, and finally placed on the ground, in a corridor, where its gait was assessed. The observer, positioned behind the bird, gently encouraged it to walk (using a stick if necessary), and recorded the gait score.

Mortality was recorded daily in each room and included both birds found dead and those culled for welfare reasons (e.g., very small size, leg deformities, lameness, or other abnormalities likely to cause suffering).

*Litter Quality.* On D36, litter quality was assessed in each room by a single trained assessor using the protocol Welfare Quality® (2009). Visual scoring was performed in five different areas per room, using the following scale: score 0 = completely dry and flaky; score 1 = dry, but not easy to move with foot; score 2 = foot imprint visible and will form a ball if compacted, but the ball does not stay together well; score 3 = sticks to boots and readily forms ball if compacted, score 4 = sticks to boots once the (compacted) crust is broken. The final score for each room was expressed as the mean of the five area scores. In addition, 18 core litter samples (approximately 10 cm in diameter) were collected from various locations within each room and pooled. A 1 kg subsample was then weighed, dried for 24 hours at 70 °C, and reweighed to determine dry matter content.

### Statistical analysis

Descriptive analyses were conducted to examine the use of enrichment items as a function of age. The number of behavioral events, specifically “Walking”, “Running”, “Crossing”, “Foraging”, and “Dustbathing”, as well as the number of “Standing” chickens were expressed as a percentage of the number of birds present in the observed area. This reference number was defined as the average of the bird counts at the beginning and end of each 3-minute observation period.

Data from the NO and H reactivity tests were analyzed separately from each session. For these tests, as well as for locomotion, foraging, dustbathing, mortality and litter quality, the statistical unit was the room. In contrast, for detour test, NE test, body weight, leg health and breast cleanliness, the statistical unit was the individual bird.

Statistical analyses were performed using R version 4.0.3 and R-Studio (R Core Team, 2023).

Prior to modeling, data and residuals were tested for normality using the Shapiro–Wilk test, and for homogeneity of variances using the Bartlett test.

A mixed linear model was applied using the *lmer* function from the *lme4* package (version 1.1-35.5), with treatment as a fixed effect. For locomotion, foraging and dustbathing data, day was included as a random effect nested within room. The number of chickens approaching or pecking at the NO or H were modeled with scans nested within rooms as random effects. A normal error distribution was assumed for all response variables. Results are presented as least squares means ± standard errors.

Some variables did not follow a normal distribution, even after logarithmic or square root transformation. Although generalized linear mixed models (GLMMs) can accommodate non-normally distributed data and account for random effects, we opted to use Mann–Whitney U tests for certain variables (e.g., latencies, mortality, and litter quality) due to their distributional characteristics and the simplicity of the experimental design for these specific outcomes. In particular, these variables were not associated with repeated measures limiting the relevance of random effects modeling.

Mann-Whitney U tests (*wilcox.test, stats* package version 4.4.2) were used to compare C and E treatments for the detour test (latency in leaving the U detour area and latency in reaching conspecifics), for the NE test (latency to walk, latency to change zone, number of zone changes, and number of explored zones), for the H and NO tests (all latencies), for litter quality, and for mortality. These data are expressed as medians with interquartile ranges [Q1, Q3].

A Student’s *t*-test was realized to compare body weight between C and E broilers (*t.test, stats* package version 4.4.2).

For categorical data, such as the number of birds scoring 0 for leg health and breast cleanliness, as well as proportions derived from emotional state tests (in the detour test, proportions of birds leaving the U detour area, reaching congeners, vocalizing; in the NE test, proportions of birds walking, changing zones, and vocalizing), comparisons compared between treatments using Chi-square test (*chisq.test,* package *stats* version 4.4.2).

Statistical analysis of the microbiota data was performed using *Easy 16S* (Midoux et al., 2024); relying on the *Phyloseq* R package and tightly integrated to the *FROGS* pipeline. All analyses, except for differential abundance, were conducted on rarefied counts, using the sample with the lowest abundance (i.e., 4,610 sequences).

Depending on whether parametric assumptions were met, comparisons of the relative abundance of different phyla between C and E groups were performed using either *t*-tests or Mann-Whitney U tests.

Alpha diversity was estimated using observed richness, Chao1, Shannon and InvSimpson indices. Comparisons between treatments were made using Mann–Whitney U tests.

Beta diversity was assessed using Bray-Curtis distances and visualized via Principal Coordinate Analysis (PCoA). The effect of treatment on microbial community structure was tested using permutational multivariate analysis of variance (PERMANOVA) with the adonis function from the *vegan* R package.

Differential abundance analysis was performed on non-rarefied ASV abundances using the *DeSeq2* R package. The analysis employed the *postcounts* size factor estimation method and Benjamini–Hochberg correction for multiple testing to identify ASVs with significantly different abundances between treatments.

For all analyses, differences were considered significant when *P* ≤ 0.05, and trends were discussed when *P* < 0.1.

Data Availability

The raw sequence data is available from NCBI SRA database with accession number PRJNA1246149. All other data can be provided upon request.

## Results

### Enrichment use

***Use of Pecking Blocks in E and C Rooms*.** Pecking blocks were used for pecking (possibly leading to particles ingestion), scratching and perching. Of the 8 kg provided per pecking block (x 2 in C and x 3 in E rooms), an average weight of 5.7 (± 0.4) kg and 6.1(± 0.7) kg per block remained at the end of the rearing period in C and E rooms.

***Use of Dust Pan in E Rooms*.** Use of the dust pan began at D10 and increased over time (up to 6 chickens observed into the pan simultaneously at D38, and 7 perched on its side at D23). Dustbathing was regularly observed there (Supplementary Figure 1).

***Use of Perforated Dispensers in E Rooms*.** Daily measurements of the remaining quantity in the suspended pecking elements (dispensers) showed that from D13 onwards, the dispensers were refilled every 3 days on average, with some variation depending on the contents (Supplementary Figure 2). Foraging balls placed on the floor remained intact during the first 10 days. From day 11 onwards, they were found open frequently and required almost daily refilling.

***Use of Straw Bales in E and C Rooms*.** The chickens sat alongside the bales with heavy use during the first 10 days of the experiment, with an average of 7.5 E and 12.6 C chickens per bale during this period (Supplementary Figure 3). After this initial period, the number of chickens positioned alongside the bales stabilized at approximately 3.5 per bale in E rooms and 4.8 C in C rooms.

Perching on the bales began after D10, with an average of 2.0 chickens perched simultaneously per bale (maximum observed: 5), in both E and C rooms (Supplementary Figure 4).

The rate of bale degradation differed between treatments: bales in the C rooms wore down more quickly and had to be replaced from D16, whereas those in the E rooms remained intact until D22.

***Use of Platforms in E and C Rooms*.** Platforms (in both the E and C rooms) were used by the chickens as of D1, with approximately 15 chicks observed on the ramps. Between D2 and D15, the number of chickens on the platforms increased similarly in both E and C rooms, exceeding 100 chickens per room by one week of age. This corresponded to approximately 25 chickens per m² on C platforms and 14 chickens per m² on E platforms), despite the larger surface area available in the E rooms.

After D15, the number of chickens on the platforms plateaued in the C rooms at around 120 chickens (30 chickens per m², with an average weight of 443 g, i.e. 13.3 kg per m²), indicating fully occupancy. In contrast, the number continued to increase in E rooms, reaching full occupancy after D20, with approximately 133 chickens (19 chickens per m², with an average weight of 700 g, i.e. 13.3 kg per m²). From that point onwards, platforms in both platforms remained fully occupied until the end of the rearing period. During this time, the number of chickens on the platforms decreased as their body weight increased.

***Use of Linear Perches in E Rooms.*** In the E rooms, linear perches were used from D6, with the number of chickens perched increasing with age, up to D30, then gradually declining (Supplementary Figure 5). The maximum was 7 chickens per 1.50 m perch (i.e. 21 cm per chicken) observed at D20 and D28, while the average ranged around 4 birds per perch between D20 and D30, and dropped to 2.9 thereafter.

***Use of Dividers in E Rooms*.** The number of chickens sitting alongside the dividers increased over the first 15 days, reaching an average of 84 per room, with a maximum of 31 birds observed per divider. After this period, the number of birds decreased, although the area remained similarly occupied.

### Locomotion, foraging and dustbathing in non-enriched / empty areas

**Fig. 2:**
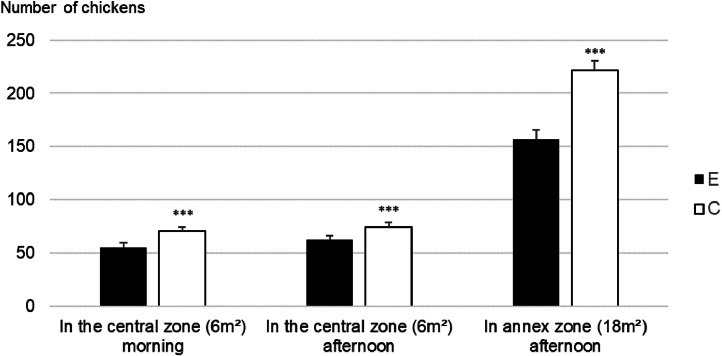
Number of chickens in the observations areas (central and annex zones) according to treatment (E: complex enriched environment, C: control environment), Statistical significance: * *P* < 0.05; ** *P* < 0.01; ***P < 0.001.

**Fig. 3:**
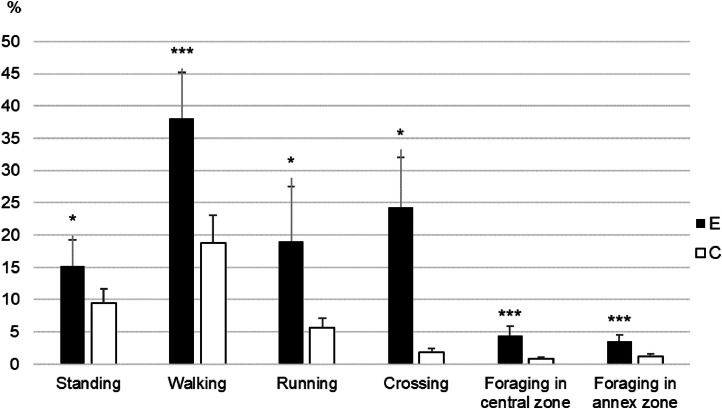
Number of standing chickens as a percentage of the chickens present in the area; and occurrences over a 3-minute period of walking, running, foraging and dustbathing relative to 100 chickens present according to treatment (E: complex enriched environment, C: control environment), Statistical significance: * *P* < 0.05; ** *P* < 0.01; ***P < 0.001.

Significant differences between E and C treatments are shown in Fig. 2, Fig. 3. All data are in Supplementary Table 1. Observation areas of E rooms (central or annex zones) were always less occupied than C corresponding ones (*P* < 0.001, Fig. 2). The percentage of Standing chickens was higher in E than in C (Fig. 3, P = 0.022). More Walking, Running, and Crossing events were observed in E treatments than in C treatments (*P* < 0.001, *P* = 0.035, *P* = 0.013 respectively). There were more occurrences of E chickens than C chickens Foraging (in the 2 observed areas, *P* < 0.001), but there was no difference in the number of Dustbathing events.

### Emotional state: novel environment, reactivity and detour tests

NE Test. No significant difference was highlighted between groups on the observed indicators of the NE test: of the 60 chickens tested per treatment, 34 E and 30 C chickens changed zone (*P* = 0.464) and 50 E and 45 C vocalized (*P* = 0.261). The number of animals walking was higher in the E treatment than in the C treatment, with a statistical trend toward significance (53 vs. 45 animals; P = 0.059). The times to the first walking event and the first zone change were not significantly different between E and C chickens: 12 [1; 46.5] s for E chickens vs. 10 [1.5; 33] s for C chickens (*P* = 0.494) for the first walking event and 70 [14; 110] s for E chickens vs. 45 [8; 79] s for C chickens (*P* = 0.419) for the first zone change. The number of explored zones was not significantly different between E and C chickens (1.0 [0.0; 2.8] and 0.5 [0.0; 2.8] respectively, *P* = 0.705), nor was the number of zone changes (1.0 [0.0; 3.8] and 0.5 [0.0; 3.8] respectively, *P* = 0.630).

**Fig. 4:**
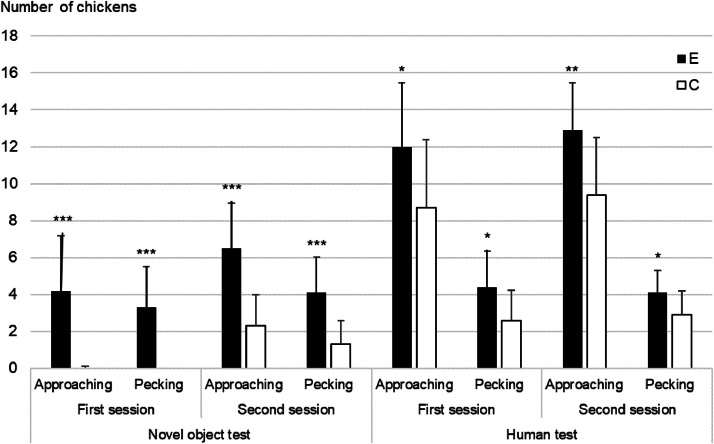
Number of chickens approaching or pecking a novel object or an unfamiliar human according to treatment (E: complex enriched environment, C: control environment), during the 2 sessions of reactivity tests. First test session: D29 for novel object test, D31 for human test, Second test session: D36 for novel object test, D38 for human test, Statistical significance: * *P* < 0.05; ** *P* < 0.01; ***P < 0.001.

Reactivity Tests. Significant differences between E and C treatments are shown in Fig. 4. All data are in Supplementary Table 2. More E chickens approached and pecked at the NO and the human than C chickens, both during the first and second sessions (*P* < 0.001 in NO test, *P* < 0.05 in Human test, Fig. 4). Latencies were not significantly different; however, there was a trend for E chickens to approach the human more quickly than C chickens (*P* < 0.1, Supplementary Table 2).

Detour Test. On the 60 chickens tested per treatment, a total of 48 E chickens left the U detour area vs. 37 C chickens (*P* < 0.001). The same number (28) E and C chickens reached the congeners. A total of 19 E chickens vocalized vs. 31 C chickens (*P* = 0.004). The latencies to leave the U detour area or to reach the congeners were both not significantly different between E and C chickens (48 [23, 162] s vs. 240 [33, 300] s respectively to leave the U detour area, *P* = 0.456, 300 [106, 300] s vs. 300 [95, 300] s respectively to reach the congeners, *P* = 0.768).

### Health and growth performances

**Fig. 5:**
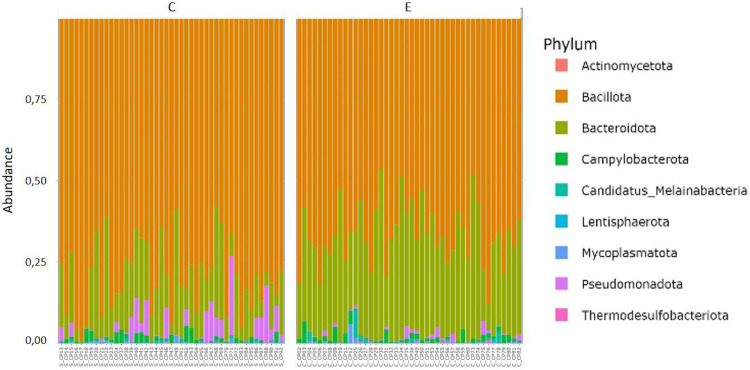
Relative abundance of the nine main phyla detected in cecal microbiota. Each bar represents a sample from the control (C) or the enriched complex environment (E) groups.

Microbiota. Chicken cecal microbiota was analyzed using 16S rRNA sequencing. After processing the sequences, 778,770 sequences were obtained (with an average number of 7963 sequences per sample) clustered in 533 ASVs. The bacteria present in the cecum of samples from both groups were mainly assigned to the phyla Bacillota and Bacteroidota representing more than 95 % of the relative abundance, and Pseudomonadota, Campylobacterota (more than 3.5 % of the relative abundance). Other minor phyla such as Actinomycetota, Candidatus_Melainabacteria, Lentisphaerota, Mycoplasmatota, and Thermodesulfobacteriota were also present (Fig. 5 and Supplementary Table S1). The five major cecal genera were Faecalibacterium, Barnesiella, Alistipes, Mediterraneibacter, and Lactobacillus (Supplementary Table S1).

**Fig. 6:**
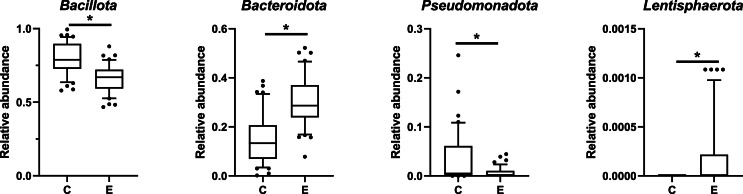
Bacterial phyla presenting significantly different relative abundances (*P* < 0.05) between C and E broilers (C: control environment, E: complex enriched environment). A t-test or Mann-Whitney U tests were used depending on whether the distribution met the parametric assumptions.

Different relative abundances were observed for four bacterial phyla between the two groups as presented in Fig. 6: relative abundance of the Bacillota and Pseudomonadota phyla were significantly higher in C chickens than in E chickens and the contrary was observed for the Bacteroidota and Lentisphaerota phyla.

No significant differences (*P* > 0.05) were observed regarding alpha diversity metrics related to species richness (observed ASVs and Chao1) and evenness (Shannon and invSimpson indices) (Supplementary Table S2).

**Fig. 7:**
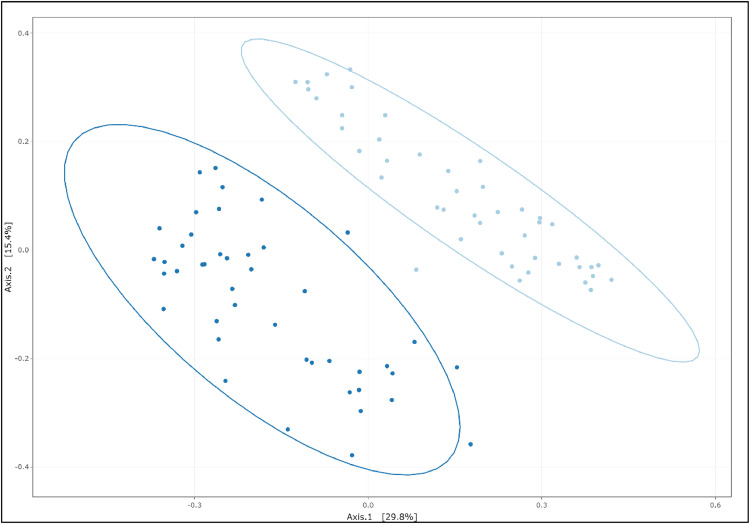
Principal coordinate analysis (PCoA) of Bray-Curtis dissimilarity of cecal microbiota from broilers reared in a control environment (C) (light blue) and a complex, enriched environment (E) (dark blue).

The beta diversity ordination plot between the two groups evaluated using Bray-Curtis dissimilarity revealed that samples from E chickens clustered separately from samples from C chickens (Fig. 7). PERMANOVA analysis showed a significant effect of the rearing environment on microbiota beta diversity (*P* < 0.001) and results indicate that 22 % of the total variance in distances can be explained by the environment.

A differential abundance analysis (using DeSeq2) was also performed to identify the ASVs contributing to the differences observed between the two groups. This analysis identified 198 ASVs that were significantly different between the two groups; 92 were significantly more abundant in E chickens (Supplementary Table S3). Table 3 focuses only on the number of 91 differentially abundant ASVs with a multiplicative factor of 4 (Log2 fold change >2 or -2) between the two groups according to their taxonomic distribution. The majority of the 91 ASVs identified belonged to the *Bacteroidota* (67 %; 61/91 ASVs) and *Bacillota* (30 %; 27/91 ASVs) phyla. Differentially abundant ASVs of the phylum *Bacteroidota* were mainly associated with E broilers, with 53 ASVs being more abundant in the E group. In particular, *Barnesiella, Alistipes, Rickenella, Odoribacter, Coprobacter* and *Bacteroides* genera were significantly more abundant in E broilers. In E chickens, differentially abundant ASVs representative of the phylum *Bacillota* belonging to the genera *Alkalibacter, Faecalibacterium*, and *Phascolarctobacterium* and one ASV belonging to the genus *Victivallis* from the *Lentisphaerota* phylum were also identified.

**Table 3:** Number of amplicon sequence variants (ASVs) significantly more abundant in group C or group E (only ASVs presenting a log2-fold change of > 2 or > -2 are presented), (E: complex enriched environment, C: control environment).

Phylum	Class	Order	Family	Genus	C	E
*Bacillota*	*Bacilli*	*Lactobacillales*	*Lactobacillaceae*	*Lactobacillus*	4	0
	*Clostridia*	*Eubacteriales*	*Christensenellaceae*	*Guopingia*	3	0
			*Eubacteriaceae*	*Alkalibacter*	0	1
			*Multi-affiliation*	*Multi-affiliation*	1	0
			*Oscillospiraceae*	*Acetivibrio*	1	0
				*Faecalibacterium*	4	3
				*Multi-affiliation*	1	0
				*Subdoligranulum*	3	0
			*Peptostreptococcaceae*	*Romboutsia*	1	0
			*Unassigned*	*Gemmiger*	1	0
			*Vallitaleaceae*	*Vallitalea*	1	0
	*Negativicutes*	*Acidaminococcales*	*Acidaminococcaceae*	*Phascolarctobacterium*	0	3
*Bacteroidota*	*Bacteroidia*	*Bacteroidales*	*Bacteroidaceae*	*Bacteroides*	1	2
			*Barnesiellaceae*	*Barnesiella*	0	21
				*Coprobacter*	0	1
			*Odoribacteraceae*	*Odoribacter*	0	2
			*Rikenellaceae*	*Alistipes*	2	17
				*Millionella*	5	0
				*Multi-affiliation*	0	8
				*Rikenella*	0	2
*Lentisphaerota*	*Lentisphaeria*	*Victivallales*	*Victivallaceae*	*Victivallis*	0	1
*Pseudomonadota*	*Gammaproteobacteria*	*Enterobacterales*	*Enterobacteriaceae*	*Multi-affiliation*	1	0
				Shigella	1	0

On the contrary differentially abundant ASVs of the phylum *Bacillota* were mainly associated with C broilers, with 20 ASVs being more abundant in the C group belonging to the genera *Lactobacillus, Guopingia, Faecalibactium, Subdoligranulum, Rombutsia, Gemmiger*, and *Vallitalea*. Differentially abundant ASVs representative of the phylum *Bacteroidota* belonging to the genera *Bacteroides, Alistipes*, and *Millionella* and two ASVs belonging to the *Enterobacteriaceae* from the *Pseudomonadota* phylum were also identified in C chickens.

Mortality, Body Weight, Leg Health, and Breast Cleanliness. No differences were observed between E and C groups in terms of mortality, body weight, and gait score (Supplementary Table 4). Only one broiler chicken was affected by pododermatitis, however, in both groups, broilers were largely affected by hock burns, with no difference between groups. Cleanliness score was 0 in more E chickens than C chickens (57 % vs. 16 % respectively, *P* < 0.001).

Litter Quality. Litter quality was not different between the two treatments (Supplementary Table 4).

## Discussion

Environmental enrichment is known to improve the welfare of broilers by enabling them to adopt a more species-specific behavioral repertoire (Riber et al., 2018). Previous studies have demonstrated that broilers engage with various enrichments such as straw bales (Mocz et al., 2022), perches (Ventura et al., 2012), dustbathing substrate (Baxter et al., 2018), and platforms (Norring et al., 2016) throughout the rearing period. In our study, Redbro broilers similarly interacted with all types of enrichment provided, especially when offered in large quantities and with sufficient diversity, though intensity and duration of use varied throughout the rearing period.

- For instance, the regular decrease in the quantity of material in the suspended pecking elements and foraging balls, particularly after D10 to 13, suggests that the chickens actively interacted with these devices. The need for refilling regularly indicates sustained interest and use, which may reflect the increasing exploratory behavior of the birds as they aged. This suggests a growing exploratory motivation with age, possibly linked to both physical development and environmental familiarity. Such age-related changes in enrichment use have been reported in other studies and may reflect the maturation of pecking and foraging behaviors (Riber et al., 2018).
- Similarly, the progressive use of the dust pan, with dustbathing observed from D10 onwards, indicates that the birds gradually engaged in comfort behaviors as they aged. The presence of multiple birds simultaneously using or perching on the pan further supports its attractiveness as a multifunctional enrichment.
- The straw bales were heavily used during the first 10 days, especially for sitting alongside, then progressively for perching. The faster degradation in C rooms compared to E rooms may reflect a lack of bales available.
- The early and sustained use of platforms by broilers indicates a strong motivation for elevated resting areas. The fact that platforms reached full occupancy, earlier in C rooms than in E rooms, suggests that the available space was insufficient, especially as birds grew. In both treatments, occupancy stabilized at around 13.3 kg/m², implying a physical or behavioral threshold. The delayed saturation in E rooms, which had larger platforms, highlights the importance of providing adequate surface area to support continued use and welfare benefits throughout the rearing period.
- The increasing linear perch use from day 6 to day 30 indicates that broilers in enriched environments are motivated to engage in elevated resting when suitable structures are available. The decline in use after day 30, despite continued availability, likely reflects physical limitations due to weight gain, making perching more difficult or uncomfortable. The drop to an average of 2.9 birds per perch supports this. These findings emphasize the need to adapt perch design to the birds’ growth, ensuring usability throughout rearing by adjusting height, width, and stability to accommodate increasing body mass.
- The initial increase in chickens resting along dividers suggests these structures offered a preferred resting area early on, likely due to perceived shelter or social comfort. The subsequent decline in bird numbers, despite similar area occupancy, may reflect space constraints as birds grew. This highlights the need to consider growth-related changes in space use when designing or evaluating enrichment structures.
- Finally despite visible interaction, the limited material loss of pecking blocks suggests moderate engagement or slow wear, possibly due to block composition or bird behavior.

Overall, these observations highlight the importance of offering diverse enrichment types that support natural behaviors at different stages. Usage patterns suggest that some enrichments (e.g., dust pans, dispensers) gain relevance over time, while others (e.g., straw bales) are most effective early in the rearing period. Material durability and accessibility also influence long-term engagement. Because the enrichments used here are available for commercial use, the present results could be extrapolated to commercial farms. The effect of the visual markers on the birds was not analyzed. However, it would be interesting to explore whether they really help the birds to locate themselves more easily in their environment, as they could easily be implemented on commercial farms at no extra cost.

Despite the high number of enrichments in E rooms, no signs of overcrowding or increased density were observed in the unenriched (“empty”) areas. On the contrary, although the number of animals per pen was identical, the central zone and annex zones were less densely populated in E rooms (9 birds/m²) compared to C rooms (12 birds/m²). This difference likely reflects the birds’ attraction to enriched zones: E birds actively engaged with specific features, such as perching on platforms (first and second tiers) and bales, exploring materials, and resting near dividers or under the platform. As a result, they redistributed themselves more evenly across the space, avoiding the empty zones. This finding aligns with Bach et al. (2019) who reported that enrichments enabling elevated resting reduced floor stocking density, potentially contributing to improved welfare by minimizing crowding and promoting behavioral diversity.

### Locomotion, foraging, and dustbathing

Compared to C chickens, E chickens were more physically active: they were observed 1.6 times more Standing, 2 times more Walking, 3.4 times more Running, and 13.5 times more Crossing the central zone. Similarly, a previous study showed that locomotion can be increased by providing bales of straw to fast-growing standard broilers (Kells et al., 2001). In this Kells’s study, control groups were housed in commercial farms without any enrichment, whereas our control groups (C) were raised with few enrichments. Our study provides novel insights by showing that not only the presence of enrichment, but also the spatial differentiation of areas within the room, can stimulate locomotor activity in fast-growing broilers. This can be due to the lower density in the empty areas, and perhaps due to the stimulation of birds moving between areas with different characteristics. It is also possible that the environmental enrichment elements placed in the E rooms limited the remaining space available to carry out these locomotor behaviors, which were then observed to a greater extent. In the E rooms, different enrichment areas were thus more related to specific behaviors, and the observation areas became dedicated to locomotion.

E chickens were also observed foraging more frequently than C chickens, even in areas not dedicated to exploration/foraging such as the central zone (5.5 times more in the central zone, 2.9 times more in the annex zone). Environmental enrichment, including litter substrate (Monckton et al., 2020) and other objects, higher space allowances (Bach et al., 2019), and elevated structures (Dawson et al., 2021), has been shown to promote foraging behavior (Riber et al., 2018). In our study, litter may have been more available due to the lower density of the observed areas, thereby promoting foraging. It is also possible that the greater exploration observed in the E rooms was associated with greater locomotion, and/or vice versa. Finally, E chickens were accustomed from an early age to an environment offering ample opportunities for exploration. Having been exposed to a variety of stimuli, they were likely more inclined to express exploratory behavior consistently across contexts compared to C individuals. This interpretation aligns with findings from de Jong et al. (2022), who demonstrated that early-life environmental conditions can have lasting effects on behavioral expression and welfare in broiler chickens, particularly through mechanisms involving habituation, coping strategies, and neurodevelopment.

Observations of dustbathing did not differ between E and C treatments in observed areas, although E chickens were observed dustbathing in the dustpans. The low frequency of dustbathing is often a reason that no differences are observed in such an experiment. Dustbathing may also depend on other factors, such as the quality of the litter, which was the same in both treatments. In fact, litter quality proved to be in fairly good condition in both treatments, perhaps explaining why it was not affected by more foraging behaviors or lower density in E treatments. This satisfactory litter quality probably led to the near absence of pododermatitis. However, a large majority of broilers were affected by hock burns, without distinction between treatments. This seems to be linked with others factors than litter quality only, and has multifactorial origins (genetic, growth rate, body weight (Kwon et al., 2024)).

Finally, even if E broilers were more active, body weights were similar between E and C treatments, but activity level can explain the better breast cleanliness in E broilers in our study.

### Emotional state

A complex and enriched environment stimulates birds’ agency, by offering them choice and control over their environment. It may contributes to a positive emotional state along with improved cognitive abilities. Moreover, in captive environments, birds may experience fear in front of new resources or management procedures (e.g.: presence an unknown human, new enrichment, new noise). A complex and enriched environment may lower the fear and stress responses of the birds. It was therefore necessary to measure whether a complex enriched environment leads to lower fear levels in chickens. Our results support this hypothesis: reactivity tests (NO and H) revealed that more E chickens approached and pecked at novel object or unfamiliar human than C chickens, despite the lower bird density in the observed area of the E treatment. Previous studies have shown that exposing chickens to a variety of stimuli can reduce their tendency to avoid or reject novel objects (Jones and Waddington, 1992). However, interpreting NO and H tests remains challenging, as both fearful and indifferent groups of animals may refrain from approaching novel object or unfamiliar human (Forkman et al., 2007). In our study, the lower number of broilers approaching the NO novel object or unfamiliar human in the C groups may reflect higher fearfulness, accompanied by a general lack of interest in the C group. On the contrary, the behavior of the E chickens suggests lower fear responses and/or greater curiosity, indicating a stronger motivation to explore their environment.

However, when tests were carried out outside the home environment and social group, such as the NE test, differences between treatments were less apparent. Apart from a tendency for more walking in the E treatment (77 % vs. 75 % in C), none of the other indicators observed were significantly affected by treatment. This contrasts with findings by Jones and Waddington (1992), where reluctance to enter an unfamiliar and exposed area, silence and inactivity were all reduced in chicks reared in an enriched environment. In our study, the lack of clear differences may be explained by the strong social reinstatement motivation triggered by isolation, which could have overridden fear-related responses. The NE test is known to reflect a conflict two different motivational states: fear (e.g., freezing, silence) and social reinstatement motivation (e.g., vocalizing, movement) (Suarez and Gallup, 1981; Marx et al., 2001; Forkman et al., 2007). Since over 75 % of birds vocalized and more than half changed zones, it is likely that social motivation dominated, potentially masking treatment effects. Finally, the absence of differences may reflect the fact that NE tests and reactivity tests assess different emotional or cognitive processes, and/or that stress from isolation, especially in birds reared in large groups, may have reduced the test’s sensitivity to enrichment effects.

The detour test also yielded informative results. Fewer than half of the broilers successfully reached their conspecifics, which may reflect either limited cognitive ability or, more likely, low social motivation, as most birds remained seated during the test. This explanation is supported by the absence of locomotor issues (no pododermatitis, low gait scores). Interestingly, these findings are consistent with Wichman et al. (2007) who reported a 44 % success rate in 4-day-old chicks, when social motivation is known to be high.

In our study, no significant difference was observed between E and C birds in reaching conspecifics, but E birds were more likely to leave the U-shaped detour area and vocalized less, suggesting greater exploratory behavior and reduced stress, in line with the reactivity test results. This may also point to enhanced spatial cognition in E birds. Previous studies have shown that early exposure to enriched environments improves spatial memory in mammals (Sneddon et al., 2000; Kobayashi et al., 2002) and spatial problem-solving in chickens (Freire and Rogers, 2005; Wichman et al., 2007). Although latency differences were not statistically significant due to high variability, the trend, E birds leaving the U detour area and reaching conspecifics quicker than C birds, further supports this interpretation.

The detour test results may reflect more than just spatial cognition, they also involve social motivation and emotional reactivity. In this context, E birds may have exited the U-shaped area not solely to rejoin conspecifics, but out of curiosity or a greater drive to explore, possibly linked to lower emotional reactivity. As Forkman et al. (2007) noted, complex fear-related behaviors are rarely driven by a single emotion like fear. Instead, factors such as exploration, imprinting, coping style, habituation, and cognitive development also shape behavioral responses. Thus, the differences observed in the detour test may reflect both cognitive performance and a greater ease of exploration in E birds. Further research is needed to disentangle these influences and confirm whether enriched environments enhance spatial cognition or primarily foster exploratory tendencies.

Group-based tests proved more effective than individual ones in revealing treatment differences, likely because individual isolation and unfamiliar environments induced higher stress levels in the chickens. Such stress may obscure differences in cognition, spatial ability, and fear responses. While habituation to the test apparatus could have improved outcomes, it was not feasible due to the large sample size, short rearing period, and declining locomotor abilities over time. Therefore, emotional reactivity had to be assessed using a battery of tests that did not require prior training, since training itself can act as a form of enrichment and potentially mask treatment effects.

Overall, this study clearly demonstrates the positive impact of environmental enrichment, while also raising important questions about how best to assess these effects. In particular, it highlights the need to develop testing methods that can capture animals’ mental states within their rearing context, minimizing external stressors.

### Microbiota

Differences in cecal microbiota were observed between E and C chickens. Previous reports have revealed the impact of rearing conditions on gut microbiota (Kers et al., 2018; de Jong et al., 2022; Marcolla et al., 2023; Muyyarikkandy et al., 2023). Alpha diversity was not affected, but beta diversity was modified reflecting differences in bacterial community composition between the two groups. Differential abundance analysis identified 198 ASVs that varied between the two groups, in particular, the genera *Barnesiella* and *Alistipes* were more abundant in E chickens. Further characterization of the microbiota functions using metagenomics and metabolomics approaches is needed to understand the role of these differentially abundant ASVs between the two groups.

At the phylum level, a higher *Bacillota:Bacteroidota* ratio was observed in C chickens compared to E chickens (significantly higher abundance of *Bacillota* and lower abundance of *Bacteroidota* in C chickens). These results are in line with previous studies, with a higher *Bacillota:Bacteroidota* ratio (referred to *Firmicutes:Bacteroidetes* in the cited literature) being previously found to be associated with semi-intensively reared chickens compared with extensively reared chickens (Susanti and Christijanti, 2022). Similar results have also been observed in Dagu chickens raised in cages compared with free-range chickens (Xiang et al., 2021). Interestingly, the *Bacillota:Bacteroidota* ratio has also been associated with welfare, because the phylum *Bacteroidota* predominates in chickens with high welfare scores (Di Marcantonio et al., 2022).

Furthermore, the phylum *Bacteroidota* has been negatively associated with poultry productivity, particularly with a decrease of total feed intake and low body weight (Deryabin et al., 2024). Therefore, taken together, these studies suggest that a higher *Bacillota:Bacteroidota* ratio can improve the utilization efficiency of feed energy. However, we did not observe any difference in the growth performance of the two groups in the present study.

Alterations in gut microbiota can influence stress responses, anxiety, and behavior, suggesting that the gut microbiota may play a role in modulating behavioral traits, which could ultimately affect productivity and welfare in poultry (Chen et al., 2022; de Jong et al., 2022; Di Marcantonio et al., 2022; Kraimi et al., 2022). This study demonstrated that the rearing environment influences both the microbiota and the behavior of the animals. However, it remains difficult to determine to what extent the microbiota contributes to the behavioral and welfare differences observed between C and E chickens.

### Limitations and future directions

The current study was designed as a whole rearing system comparison: the E rooms were characterized by several different features that may affect chickens in different ways. Therefore, it is not possible to draw conclusions on a single influencing factor, such as a particular enrichment or spatial structuring element, but must be considered in its entirety as a whole complex rearing environment. The positive effects of this configuration are not only limited to a reduction in indicators of poor welfare, but also an increase in the indicators of positive welfare.

One potential limitation is the use of mixed-sex groups, which could influence behavioral dynamics. However, our study included a balanced proportion of males and females, allowing us to assess the overall impact of environmental conditions on the flock as a whole. While it is possible that males and females may express certain behaviors differently, our primary aim was to evaluate the effects of rearing conditions on group-level welfare indicators. Nonetheless, future studies could explore sex-specific behavioral responses in more detail, particularly in the context of fundamental research on individual variability.

Another factor that may influence behavioral and physiological responses is the age of the animals. In the present study, age was not included as a fixed factor in the statistical models, as our primary objective was to assess the overall impact of environmental enrichment across the rearing period. However, age-related changes in activity levels, emotional reactivity, or cognitive performance could interact with environmental conditions. Future studies could benefit from a more detailed temporal analysis or from including age as a fixed factor to better understand how responses to enrichment evolve over time. This would be particularly relevant for identifying sensitive periods during which animals may be more responsive to environmental modifications.

Finally, the behavioral observations in this study were based on 3-minute period per room. While this may appear short, each room was observed three times per day over four consecutive days, resulting in a total of 12 observation sessions per room. This repeated sampling strategy was designed to capture representative snapshots of behavior across different times of day and over multiple days, while remaining feasible within the logistical constraints of the study. Although this approach provides a robust basis for comparing treatment groups, we acknowledge that it may not fully reflect the full range of daily behavioral patterns. Future studies could complement this method with longer or continuous monitoring to gain a more comprehensive understanding of behavioral dynamics over time.

## Conclusion

In conclusion, environmental enrichment proved beneficial to the welfare of broiler chickens, offering them greater behavioral diversity, increased locomotion and foraging, and influencing microbiota composition. In addition, several indicators point to a reduced emotional reactivity, signs of improved spatial cognition, and higher ease to explore. Being less fearful and more inquisitive could indicate better stress management and greater ability to interact with objects and humans. These effects were particularly evident in group-based tests conducted within the rearing environment, highlighting the importance of context in assessing emotional and cognitive responses.

Despite the presence of numerous and sometimes overlapping enrichment elements (e.g., multiple perching options), all devices were used in varied ways throughout the day and across the rearing period, indicating that they met the chickens’ behavioral needs. The structured layout of the enriched rooms also appeared to stimulate movement between differentiated zones, contributing to increased physical activity and foraging behavior, suggesting that enrichment and a structured environment promote exploration and enhances motor skills.

Importantly, none of the health or growth performance indicators assessed were negatively affected and breast cleanliness was improved in enriched birds.

While the study revealed differences in microbiota composition between treatments, it was not designed to directly link these changes to behavioral or emotional outcomes. Future research should explore these interactions in more depth.

Overall, enriched, complex and structured rearing environments act on multiple dimensions of welfare, behavioral, emotional, and physiological, by not only reducing indicators of poor welfare, but also enhancing indicators of positive welfare. This systemic approach, tested under semi-commercial conditions, offers promising perspectives for more welfare-oriented and behaviorally adapted broiler production systems.

## CRediT authorship contribution statement

**M. Guinebretière:** Conceptualization, Formal analysis, Supervision, Writing – original draft, Writing – review & editing. **M. Guyard-Nicodème:** Data curation, Formal analysis, Writing – original draft, Writing – review & editing. **F. Mocz:** Methodology, Resources, Writing – original draft. **L. Calandreau:** Conceptualization, Validation, Writing – original draft. **A. Scheubel:** Data curation, Methodology, Resources. **J.P. Moysan:** Methodology, Resources, Writing – original draft. **M. Chemaly:** Funding acquisition, Supervision. **A. Keita:** Supervision. **L. Warin:** Conceptualization, Funding acquisition, Methodology, Project administration, Validation, Writing – original draft.

## Disclosures

The authors declare that they have no known competing financial interests or personal relationships that could have appeared to influence the work reported in this paper.

## Appendix Supplementary materials

Image, application 1

Image, application 2

## Footnotes

Supplementary material associated with this article can be found, in the online version, at doi:10.1016/j.psj.2025.105663.
