# Supplementary material for: Structuring and enriching the rearing environment in conventional broiler chicken production: effects on behavioral indicators, emotional states, and cecal microbiota composition
Source: Poult Sci. 2025 Aug 6;104(11):105663. doi: 10.1016/j.psj.2025.105663 (PMC12398803; doi:10.1016/j.psj.2025.105663)
Supplement: Supplementary file 2 [file mmc2.docx]

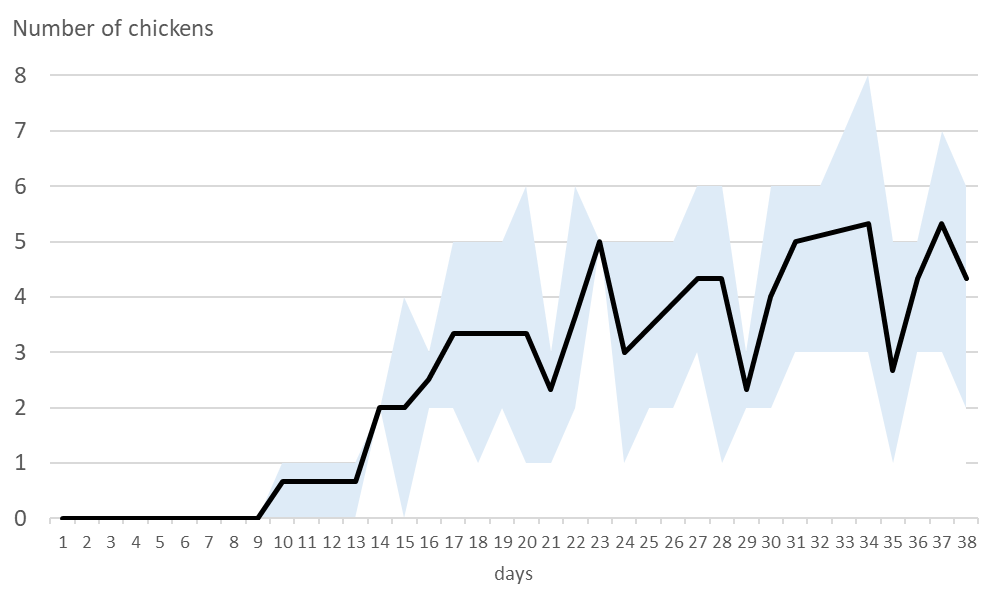


*Supplementary Figure 1: Number of chickens into and perched on one dustpan in E rooms (E: complex enriched environment, one dustpan per E room, in 3 E rooms). Mean: black line, blue: min-max observed per dustpan.*


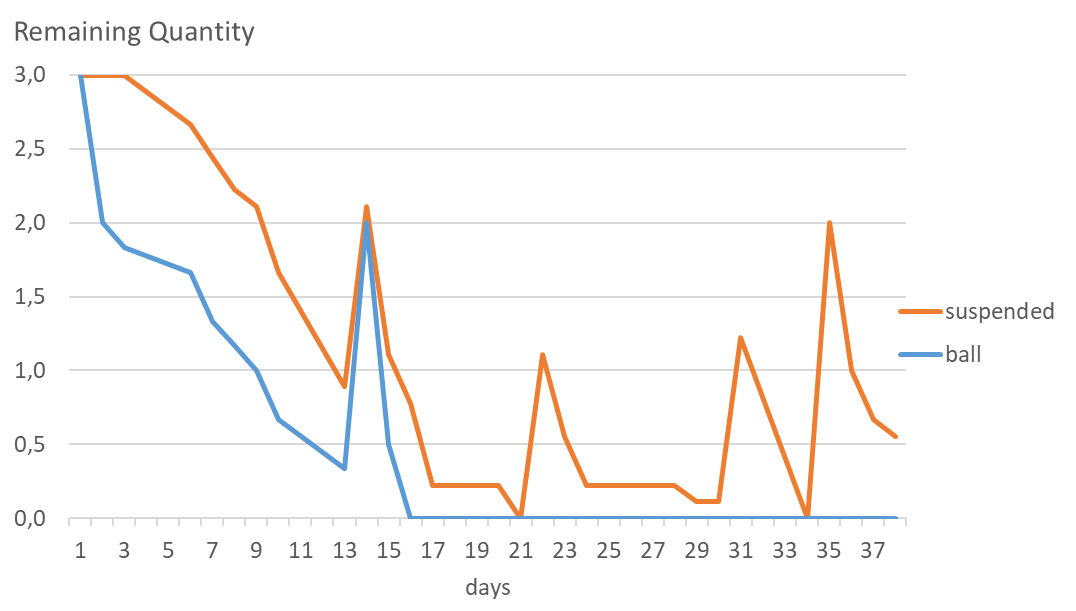


*Supplementary Figure 2: Remaining quantity of pecking material (oats, corn, wheat, or grit) in a suspended dispenser and in a foraging ball, visually scored, in E rooms (E: complex enriched environment, 3 suspended dispensers and 3 foraging balls per E room, in 3 E rooms). Scoring ranged from 0 to 3 points, the lowest score indicating empty dispenser. Dispensers were filled on D0, then whenever necessary: every 3 days on average from D13 for suspended dispensers, every day from D10 for foraging balls.*


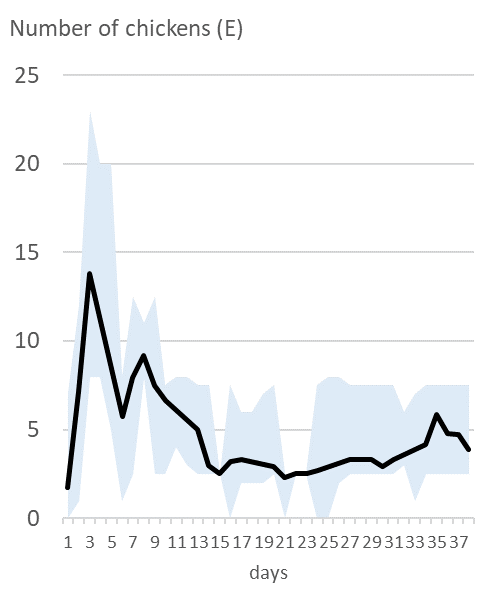

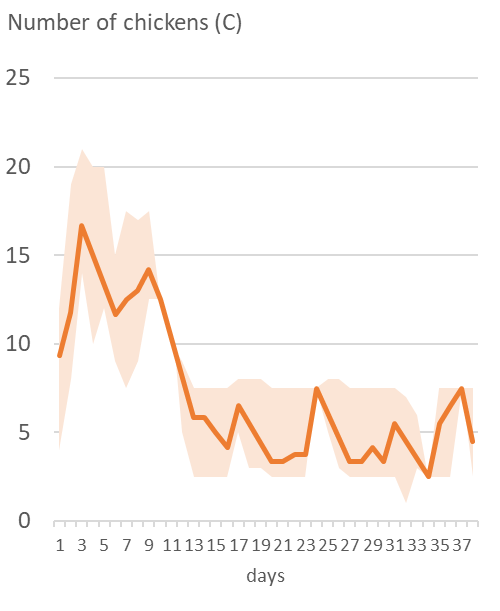


*Supplementary Figure 3: Number of chickens alongside one bale in E and C broilers (E: complex enriched environment, C: control environment, 4 bales per E room, in 3 E rooms and 2 bales per C room, in 3 C rooms). Mean: black line, blue: min-max observed per bale.*


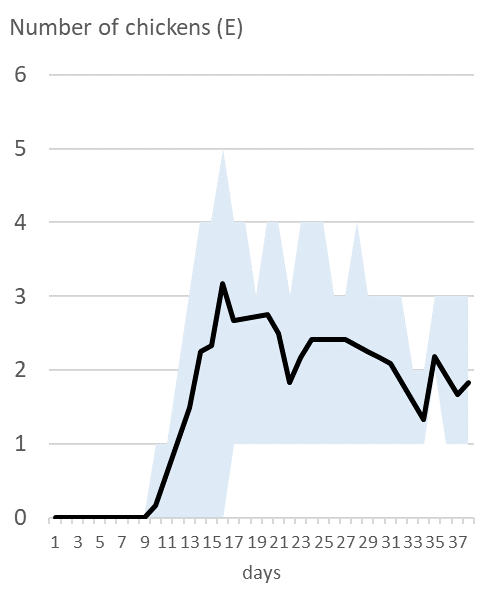

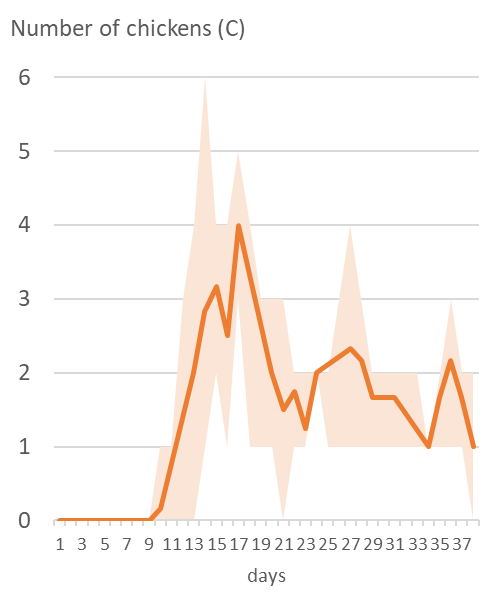


*Supplementary Figure 4: Number of chickens perched on one bale in E and C broilers (E: complex enriched environment, C: control environment, 4 bales per E room, in 3 E rooms and 2 bales per C room, in 3 C rooms). Mean: black line, blue: min-max observed per bale.*


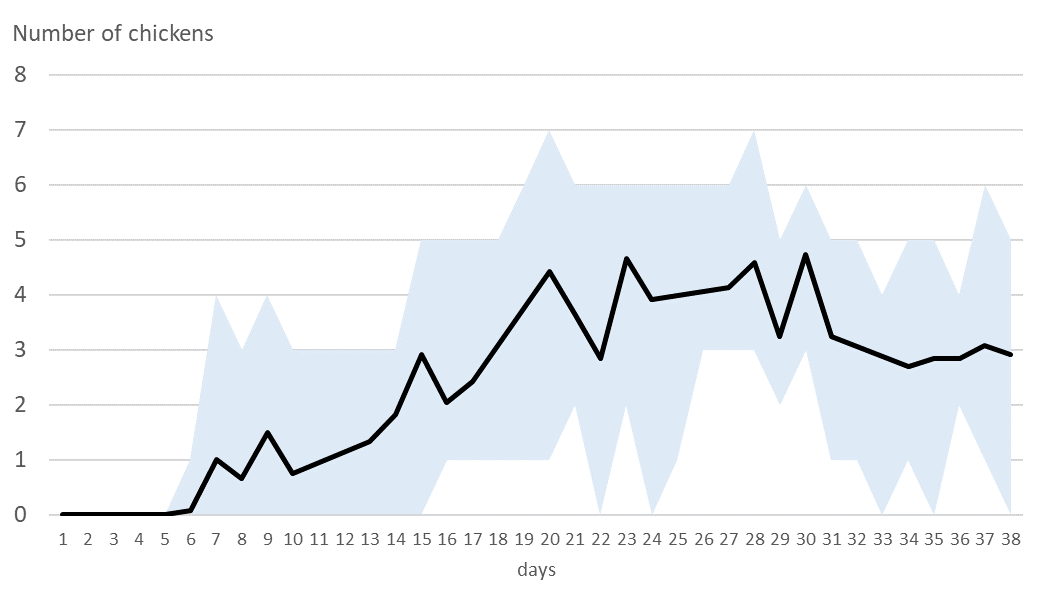


*Supplementary Figure 5: Number of chickens perched per 150 cm linear perch in E broilers (E: complex enriched environment, 4 linear perches per E room, in 3 E rooms). Mean: black line, blue: min-max observed per bale.*

*Supplementary Figure 6: Alpha diversity measure of cecal microbiota in C and E broilers (C: control environment, E: complex enriched environment).*

Supplementary Table 1 : Mean number of chickens in the observations areas (central and annex zones) (± standard error) and mean activity (± standard error) [number of standing chickens as a percentage of the chickens present in the area; occurrences over a 3-minute period of walking, running, foraging and dustbathing (see Table 1) relative to 100 chickens present] according to treatment (E: complex enriched environment, C: control environment), and significance of treatment effect (P).

| **Observations** | | **Treatment** | | ***P*** |
| --- | --- | --- | --- | --- |
|  |  | **E** | **C** |  |
| Number of chickens in central zone (6 m²) | Morning | 55.3 ± 4.3 | 70.7 ± 3.4 | < 0.001 |
|  | Afternoon | 62.7 ± 3.5 | 74.5 ± 4.5 | < 0.001 |
| Number of chickens in annex zone (18 m²) | Afternoon | 157.0 ± 8.5 | 222.0 ± 8.6 | < 0.001 |
| Percentage standing | | 15.2 ± 4.1 | 9.4 ± 2.2 | 0.022 |
| Walking | | 38.1 ± 7.1 | 18.8 ± 4.3 | < 0.001 |
| Running | | 19.0 ± 8.5 | 5.6 ± 1.5 | 0.035 |
| Crossing | | 24.3 ± 7.7 | 1.8 ± 0.7 | 0.013 |
| Foraging | in central zone | 4.4 ± 1.5 | 0.8 ± 0.3 | < 0.001 |
|  | in annex zone | 3.5 ± 1.1 | 1.2 ± 0.3 | < 0.001 |
| Dustbathing | in central zone | 1.7 ± 1.2 | 3.2 ± 1.0 | 0.217 |
|  | in annex zone | 1.2 ± 0.3 | 1.6 ± 0.3 | 0.384 |

*Supplementary Table 2: Mean (± standard error) number of chickens approaching or pecking a novel object or an unfamiliar human and corresponding median latencies [Quartile 1, Quartile 3] according to treatment (E: complex enriched environment, C: control environment), and significance of treatment effect (n = 10 scans x 3 rooms) during the 2 sessions of reactivity tests. First test session: D29 for novel object test, D31 for human test, Second test session: D36 for novel object test, D38 for human test.*

|  | **First session** | | |  | **Second session** | | |
| --- | --- | --- | --- | --- | --- | --- | --- |
|  | **E** | **C** | ***P*** |  | **E** | **C** | ***P*** |
| **Novel object test** | | | | | | | |
| Number of chickens approaching | 4.2 ± 3.0 | 0.0 ± 0.1 | < 0.001 |  | 6.5 ± 2.4 | 2.3 ± 1.7 | < 0.001 |
| Number of chickens pecking | 3.3 ± 2.2 | 0.0 ± 0.0 | < 0.001 |  | 4.1 ± 1.9 | 1.3 ± 1.3 | < 0.001 |
| Time until first chicken enters the zone (s) | 86 [81, 156] | 299 [270, 300] | 0.100 |  | 7 [5, 33] | 121 [64, 140] | 0.400 |
| Time to first peck (s) | 88 [84, 194] | 300 [300, 300] | 0.197 |  | 8 [8, 44] | 173 [96, 237] | 0.184 |
| **Human test** | | | | | | | |
| Number of chickens approaching | 12.0 ± 3.5 | 8.7 ± 3.7 | 0.014 |  | 12.9 ± 2.5 | 9.4 ± 3.1 | 0.002 |
| Number of chickens pecking | 4.4 ± 2.0 | 2.6 ± 1.6 | 0.018 |  | 4.1 ± 1.2 | 2.9 ± 1.3 | 0.020 |
| Time until first chicken enters the zone (s) | 19 [10, 33] | 37 [19, 67] | 0.825 |  | 1 [0,2] | 32 [20, 42] | 0.064 |
| Time to first peck (s) | 35 [33, 62] | 139 [97, 144] | 0.200 |  | 1 [1, 21] | 49 [42, 60] | 0.200 |

*Supplementary Table 4: Mortality at D42, physical condition at D35 (body weight and percentage of broiler chickens that were scored 0 (no disorders) for pododermatitis, hockburns, gait, breast cleanliness pododermatitis, hock burns, gait, and breast cleanliness), Litter quality score and dry matter content, according to treatment (E: complex enriched environment, C: control environment), and significance of treatment effect (*P*). Scoring ranged from 0 to 2 points for each criterion, the lowest score indicating the best condition.*

|  | **Treatment** | | ***P*** |
| --- | --- | --- | --- |
|  | **E** | **C** |  |
| Mortality D42 | 3.66% | 3.98% | 0.364 |
| Body Weight at D35 | 1777g | 1749g | 0.280 |
| Pododermatitis | 150 | 149 | 0.153 |
| Hock burns | 25% | 27% | 0.792 |
| Gait | 99% | 97% | 0.514 |
| Breast Cleanliness | 57% | 16% | 0.001 |
| Litter quality score | 1.6 | 1.5 | 0.200 |
| Litter dry matter content (%) | 58.5% | 59.8% | 0.320 |
